# Trends in causes of death among children under 5 in Bangladesh, 1993-2004: an exercise applying a standardized computer algorithm to assign causes of death using verbal autopsy data

**DOI:** 10.1186/1478-7954-9-43

**Published:** 2011-08-05

**Authors:** Li Liu, Qingfeng Li, Rose A Lee, Ingrid K Friberg, Jamie Perin, Neff Walker, Robert E Black

**Affiliations:** 1 Department of International Health, Johns Hopkins Bloomberg School of Public Health, 615 N. Wolfe Street, Baltimore, MD 21205, USA; 2 Department of Population, Family and Reproductive Health, Johns Hopkins Bloomberg School of Public Health, 615 N. Wolfe Street, Baltimore, MD 21205, USA

## Abstract

**Background:**

Trends in the causes of child mortality serve as important global health information to guide efforts to improve child survival. With child mortality declining in Bangladesh, the distribution of causes of death also changes. The three verbal autopsy (VA) studies conducted with the Bangladesh Demographic and Health Surveys provide a unique opportunity to study these changes in child causes of death.

**Methods:**

To ensure comparability of these trends, we developed a standardized algorithm to assign causes of death using symptoms collected through the VA studies. The original algorithms applied were systematically reviewed and key differences in cause categorization, hierarchy, case definition, and the amount of data collected were compared to inform the development of the standardized algorithm. Based primarily on the 2004 cause categorization and hierarchy, the standardized algorithm guarantees comparability of the trends by only including symptom data commonly available across all three studies.

**Results:**

Between 1993 and 2004, pneumonia remained the leading cause of death in Bangladesh, contributing to 24% to 33% of deaths among children under 5. The proportion of neonatal mortality increased significantly from 36% (uncertainty range [UR]: 31%-41%) to 56% (49%-62%) during the same period. The cause-specific mortality fractions due to birth asphyxia/birth injury and prematurity/low birth weight (LBW) increased steadily, with both rising from 3% (2%-5%) to 13% (10%-17%) and 10% (7%-15%), respectively. The cause-specific mortality rates decreased significantly due to neonatal tetanus and several postneonatal causes (tetanus: from 7 [4-11] to 2 [0.4-4] per 1,000 live births (LB); pneumonia: from 26 [20-33] to 15 [11-20] per 1,000 LB; diarrhea: from 12 [8-17] to 4 [2-7] per 1,000 LB; measles: from 5 [2-8] to 0.2 [0-0.7] per 1,000 LB; injury: from 11 [7-17] to 3 [1-5] per 1,000 LB; and malnutrition: from 9 [6-13] to 5 [2-7]).

**Conclusions:**

Pneumonia remained the top killer of children under 5 in Bangladesh between 1993 and 2004. The increasing importance of neonatal survival is highlighted by the growing contribution of neonatal deaths and several neonatal causes. Notwithstanding the limitations, standardized computer-based algorithms remain a promising tool to generate comparable causes of child death using VA data.

## Background

Trends in the causes of child mortality serve as important global health information to guide efforts to improve child survival [1,2]. For a low- or middle-income country (LMIC) like Bangladesh, these indicators are particularly important for assisting child health policy development and scarce resource allocation.

Child mortality rates are declining in many countries [3,4]. In Bangladesh, the under-5 mortality rate (U5MR) decreased from 148 to 52 deaths per 1,000 live births (LB) between 1990 and 2009 [4]. During the same period of time, the neonatal mortality rate also dropped from 58 to 30 deaths per 1,000 LB. As a result, neonatal deaths contributed 57% of all under-5 deaths in 2009, compared to only 39% two decades earlier. Accompanying this steady decline in child mortality is a changing distribution of child causes of deaths [1,5], which has not been well described previously.

The three verbal autopsy (VA) studies conducted with the Bangladesh Demographic and Health Surveys (BDHS) provide a unique opportunity to fill this gap [1,6,7]. The goal of the current study is to apply data from the three surveys to generate nationally representative empirical trends of child causes of death. Specifically, we aim to first develop a standardized computer algorithm to assign causes of death and then to apply the standardized algorithm to generate comparable estimates over time for causes of child death.

## Methods

### Data source and datasets

The three VA studies were conducted in 1993-1994, 1996-1997, and 2004, following the corresponding BDHS. Details of the design and implementation of each study were published elsewhere [6-8]. Briefly, all three BDHS have a multistage stratified and clustered sampling design, with a total sample size of 9,174; 8,682; and 10,500 households and 12,924 (9,174 women and 3,284 men); 12,473 (9,127 women and 3,346 men); and 15,627 (11,330 women and 4,297 men) individuals for 1993-1994, 1996-1997, and 2004, respectively. Households with under-5 deaths in the past five years were revisited for the VA studies after the main DHS was conducted. The VA interviews were performed after the completion of the 1993-1994 and 1996-1997 DHS, but were done within one day of identifying the eligible households in 2004. Standardized VA questionnaires, also including a narrative of the conditions regarding the fatal illness, were administered to primary caretakers of deceased children. Only data collected through the structured questionnaires were analyzed here. Information on 828, 678, and 587 under-5 deaths was collected in 1993-1994, 1996-1997, and 2004, respectively, which corresponds to 91%, 94%, and 99% of the eligible under-5 deaths in the three surveys.

We obtained the VA instruments and datasets from the three studies with help from colleagues at the Johns Hopkins Bloomberg School of Public Health and DHS at Inner City Fund (ICF) Macro. The 1993-1994 and 1996-1997 VA studies are nearly identical in design, but both differ from the 2004 study in many aspects. Some of the differences are summarized elsewhere [7]. In general, more symptom data were collected in 2004. In addition, different algorithms were applied when assigning causes of death in each study. In order to generate comparable trends in child causes of death, a standardized algorithm that was compatible with all three studies was developed.

### Development of the standardized algorithm

Computer-based algorithms following a hierarchical process were originally applied in all three VA studies [6-8]. We chose to develop the standardized algorithm in a similar fashion. During the development, the original algorithms applied in each study were systematically reviewed. Four aspects of the algorithms - cause categorization, hierarchy, case definition, and amount of information collected - were compared and considered in the process.

#### Cause categorization

As shown in Table 1, the same categorization was used for many causes across the three studies, including injury, neonatal tetanus, measles only, measles followed by acute respiratory infections (ARI)/diarrhea, ARI (note that the terms ARI and pneumonia are used interchangeably in this paper), malnutrition, and unidentified causes. For most of the other cause categories, the differences were trivial. For example, possible ARI, possible diarrhea, and possible ARI and diarrhea appeared as individual causes previously, but were combined with other possible serious infections in the last study.

**Table 1:** Cause categories applied in the three VA studies in Bangladesh

1993-1994	1996-1997	2004
Injury	Injury	Injury

Neonatal tetanus		

Measles only		

Measles followed by ARI/diarrhea		

ARI		

Watery diarrhea		
	
Persistent diarrhea		Diarrhea
	
Dysentery		

ARI and watery diarrhea		
	
ARI and persistent diarrhea		ARI and diarrhea
	
ARI and dysentery		

Congenital abnormality		Congenital abnormality
		
Prematurity		Premature birth/LBW
	**Early neonatal or** **pregnancy/delivery** **related**	
		Birth asphyxia
**Early Perinatal**		
		Birth injury

Possible ARI		Other possible serious infections
	
Possible diarrhea		
	
Possible ARI and diarrhea		

Malnutrition		

Not identified		

However, one important difference exists between cause categorizations across studies. In the first study, deaths occurring in the first three days of life except congenital abnormality and prematurity were collapsed to form the category of "early perinatal deaths" [8]. In the second study, all deaths occurring in the first three days, including those due to congenital abnormality, prematurity, and complications of delivery, were included in the category of "early neonatal or pregnancy/delivery related" deaths [6]. These collapsed categories masked the relative importance of individual neonatal causes. In fact, information was collected and available in both studies to assign the specific neonatal causes, but was not originally used to classify subcategories.

#### Hierarchy

**Figure 1:**
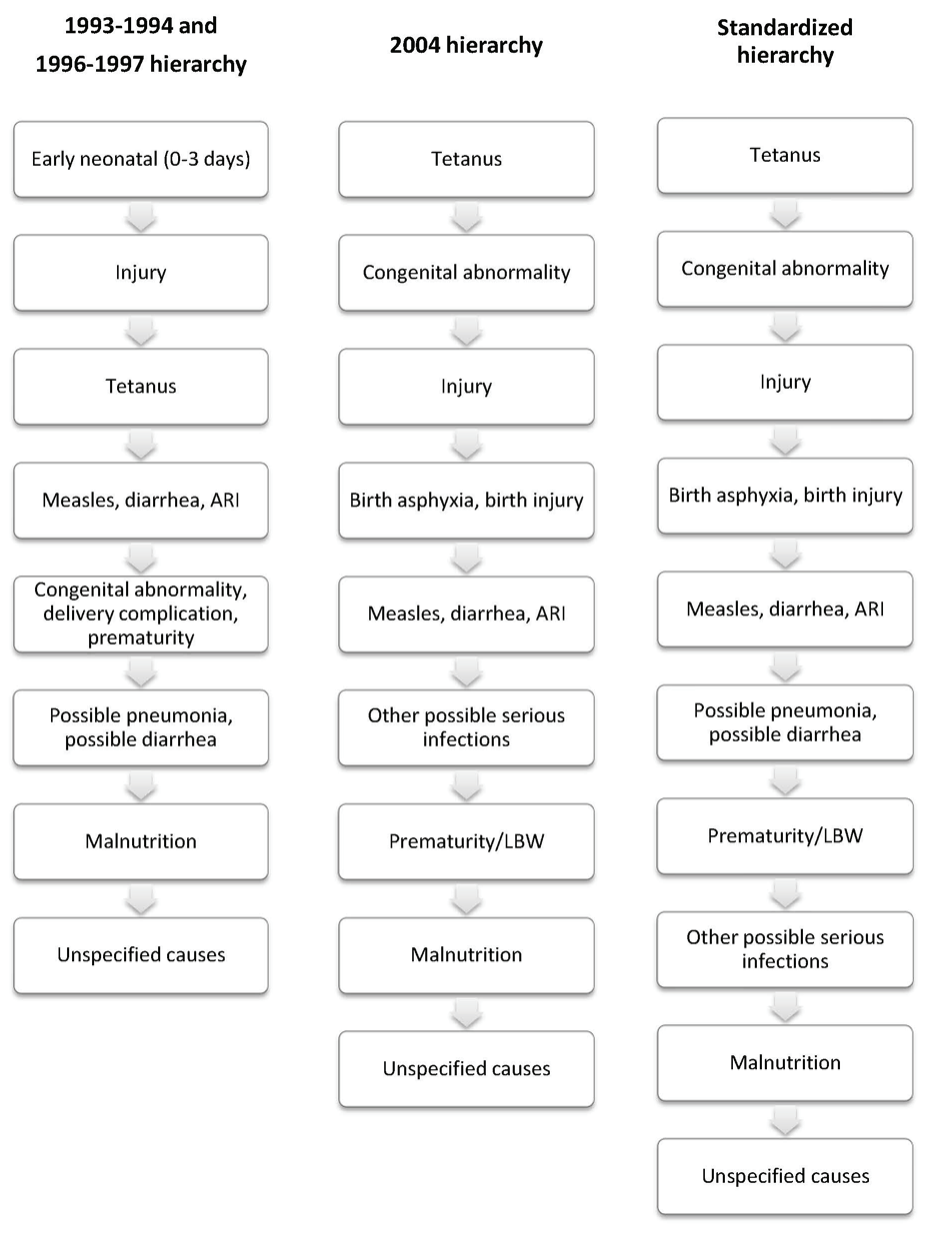
**Hierarchies applied in the three Bangladesh VA studies and the standardized hierarchy**.

The hierarchies applied in the first two VA studies are nearly identical [6,7], but are quite different from the one used in 2004 (Figure 1, left and middle columns). As discussed above, one important difference is the classification of early neonatal deaths in the first two studies. Because different cause categorizations were applied, the total number of tiers was different in the hierarchies. The same causes could also be given different priorities and assigned in different tiers. For example, neonatal tetanus was assigned in the third tier in the first two studies but in the first tier in 2004. Prematurity/LBW was assigned before possible pneumonia/diarrhea initially, but was moved to be assigned after other possible serious infections in 2004.

#### Case definition

The definitions for each cause, or the case definitions, are similar across the three studies (see Additional file 1 for details), but are slightly different for a number of causes. For example, neonatal tetanus was defined in the first two studies as deaths occurring between 4 and 14 days of life, with convulsions, and where the baby either cried normally after birth but stopped crying in the final illness or suckled normally after birth but stopped suckling in the final illness or both. In 2004, the case definition included one additional condition: i.e., the symptoms of "cried normally after birth but stopped crying" and "suckled normally after birth but stopped suckling" needed to happen not only in the terminal stage of illness, but also at least one day before the final illness. Other differences in case definitions are underscored in Additional file 1 where applicable.

#### Amount of information collected

Even if the same case definitions were applied across the three studies, the causes of deaths would not always be comparable due to the fact that different amounts of information were collected in each study. For example, the definition of other possible serious infections entails having two or more signs of serious infections. However, a different number of signs of serious infections were collected in each study. Specifically, five signs of serious infections (stopped suckling, difficult breathing, chest indrawing, convulsions, and fever) were collected in all three studies, with one more sign (stopped crying) collected in 1996-1997 and 2004, and 12 additional signs (rapid breathing, cold to touch, lethargic, unresponsive or unconscious, bulging fontanels, redness or drainage from the umbilical cord stump, skin rash with bumps containing pus, vomiting everything, stopped being able to grasp, stopped being able to respond to a voice, stopped being able to follow movements with the eyes, and stiff neck) collected in 2004 alone. As a result, a larger proportion of other possible serious infections would have been assigned in 2004 because more signs were available in that year.

#### The standardized algorithm

After systematically reviewing the original algorithms, we consider the 2004 cause categorization and hierarchy to be more logical, and developed the standardized algorithm mainly based on them. Even though some specific neonatal causes, such as birth asphyxia, were not assigned in the two earlier studies, relevant signs and symptoms were collected. This allowed us to further classify the causes of most cases originally assigned as early neonatal deaths. The final cause categories are in principle consistent with those adopted by the Child Health Epidemiology Reference Group (CHERG) [1,2,9]. Specifically, results for the following causes are presented: pneumonia, diarrhea, prematurity/LBW, birth asphyxia/birth injury, congenital abnormalities, neonatal tetanus, measles, injury, possible serious infections, malnutrition, and unspecified causes. It is noted that drowning, as an important cause among children aged 1 to 4 years, is included in injury. We also incorporated into the final hierarchy certain tiers from the 1993-1994 and 1996-1997 studies, specifically, possible pneumonia and possible diarrhea, and considered them to be meaningfully different from the remaining other possible serious infections. We promoted prematurity/LBW to be classified before the remaining other possible serious infections. The final hierarchy is shown in Figure 1 (the right column), which indicates the specific steps that were taken to assign deaths by cause in this study. We also attempted to assign meningitis in the hierarchy by applying the case definitions borrowed from the 1999 World Health Organization VA monograph [10], but were not successful due to the lack of the necessary signs of serious infections in the first two studies.

Given that our objective was to generate comparable trends in the causes of under-5 deaths, we decided to adopt case definitions that could be employed in all three studies. For example, we chose to apply the case definition of neonatal tetanus from the first two studies and ignore the additional information available in 2004 despite the fact that the 2004 definition may have higher specificity. Similarly, when deciding how much information to apply in the standardized algorithm, to serve our purpose of maintaining comparability, we chose to only include the amount of information that was available across all studies. In the case of other possible serious infections, only the five commonly available signs of serious infections were employed in the algorithm.

Some causes could occur both among neonates and children from 1 to 59 months old, such as diarrhea, possible diarrhea, ARI, possible ARI, and other possible serious infections. They were assigned in the same tier, but separately for the two age groups applying somewhat different case definitions (see Additional file 1 for details). Measles, diarrhea, and ARI were assigned at the same time, which allowed any two or all three causes to be assigned. The resulting cause categories include measles only, measles followed by ARI or diarrhea, diarrhea only, pneumonia only, and ARI and diarrhea [6-8]. When presenting the final causes, measles and measles followed by ARI or diarrhea were combined as measles. ARI and diarrhea were redistributed among ARI only and diarrhea only according to their relative importance as assigned single causes. Similar redistribution was done for possible pneumonia and possible diarrhea. Then possible pneumonia and possible diarrhea as two causes were grouped into pneumonia and diarrhea, respectively, to form the final categories of pneumonia and diarrhea. The standardized algorithm was reviewed by members of the CHERG before submission for publication.

### Sensitivity analysis and uncertainty estimation

After the standardized algorithm was finalized, it was applied to the three VA studies to obtain cause-specific fractions. The cause fractions were properly weighted to take into account oversampling of certain subgroups in the BDHS [6-8]. To reduce the influence of the hierarchy, we conducted a sensitivity analysis to first assign causes allowing multiple diagnoses without following a hierarchy. Then, among deaths with multiple causes, we applied the standardized hierarchy to determine a single cause.

To directly compare trends in each cause across time, cause-specific mortality rates (CSMR) were calculated by multiplying cause-specific fractions with U5MR. Estimates of U5MR are available from multiple sources for Bangladesh, including the DHS [7,11,12], the Inter-agency Group for Child Mortality Estimation (IGME) [4], and the Institute for Health Metrics and Evaluation (IHME) [3]. The three sets of U5MR are generally similar but larger discrepancies were observed in 2004. For the years of 1993-1994 and 1996-1997, U5MR were interpolated for midpoints between the two years in all three series accordingly. All three sets of U5MR were applied to calculate CSMR, and the results were not qualitatively different. The IGME series was presented here for demonstration since it ranked in the middle of the three sets of estimates.

When producing uncertainty estimates, to take into account the complex survey design of the studies, a bootstrap sample of clustered data was taken for each DHS survey within strata, where strata contained multiple primary sampling units. Strata with only one primary sampling unit contributing cause of death data were treated interchangeably, or as if they were in a single stratum. Because deaths were only observed at the household or secondary sampling unit, this led to a substantial estimated variability in the cause of death estimates. This uncertainty was used as a factor together with the uncertainty of the U5MR [4] to determine the total uncertainty in the CSMR. Uncertainty ranges (UR) were defined as the 2.5 to 97.5 percentiles. A two-sample bootstrap test was implemented to examine whether the differences were statistically significant between the 1993-1994 and 2004 studies. The analyses were conducted using STATA 10.0 Special Edition [13] and R [14].

## Results

### Trends in the cause-specific fractions

**Figure 2:**
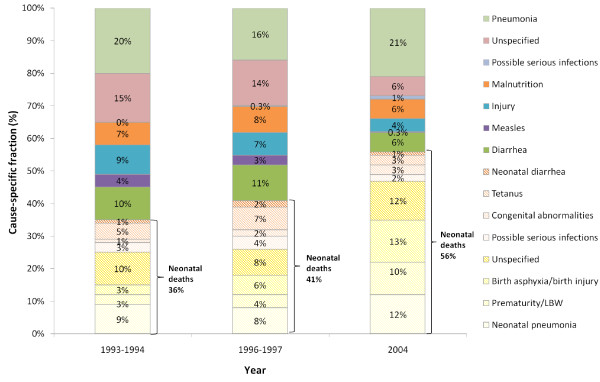
**Cause-specific fraction of deaths among children under 5 years of age in Bangladesh, 1993-1994, 1996-1997, and 2004**.

In Bangladesh, the under-5 mortality rate dropped from 128 deaths per 1,000 live births (LB) in 1993-1994 to 110 in 1996-1997, and then to 70 per 1,000 LB in 2004 [4]. During the same time period, the neonatal mortality rate (NMR) declined from 53 to 49 and then to 36 deaths per 1,000 LB. Corresponding to the reduction in under-5 mortality, the proportion of neonatal deaths increased significantly from 36% (UR: 31%-41%) to 41% (37%-46%) and then to 56% (49%-62%) in the three VA studies (Figure 2, also refer to Additional file 2 for a complete list of uncertainty estimates).

Neonatal pneumonia remained one of the most important causes across time, contributing to 9% (6%-12%), 8% (5%-11%), and 12% (9%-17%) of under-5 deaths in 1993-1994, 1996-1997, and 2004, although the change is not statistically significant. The relative importance of birth asphyxia/birth injury increased steadily and significantly, claiming 3% (2%-5%), 6% (4%-9%), and 13% (10%-17%) of deaths among children younger than 5 in the three studies. Similarly, the proportion of deaths due to prematurity/LBW increased significantly from 3% (2%-5%) and 4% (2%-7%) to 10% (7%-15%) during the same period of time.

Among children aged 1 to 59 months, pneumonia remained the top killer over the decade, claiming 16% to 21% (1993-1994: 20% [16%-25%]; 1996-1997: 16% [12%-20%]; 2004: 21% [16%-26%]) of lives of children under 5 in Bangladesh. The contribution of diarrhea decreased from 10% (6%-13%) and 11% (7%-14%) in the first two studies to 6% (3%-9%) in 2004. The cause-specific fraction of measles declined from 4% (2%-6%) to 0.2% (0%-0.9%) in 1993-2004. Injury and malnutrition also posed major threats to child survival, contributing 4% to 9% (1993-1994: 9% [5%-13%]; 1996-1997: 7% [4%-9%]; 2004: 4% [2%-8%]) and 6% to 8% (1993-1994: 7% [5%-10%]; 1996-1997: 8% [5%-11%]; 2004: 6% [3%-10%]) of total under-5 deaths, respectively. None of the changes in cause-specific fractions are significant among 1- to 59-month-olds except those due to measles and injury.

Among all children under 5, pneumonia was responsible for 24% to 33% (1993-1994: 29% [25%-34%]; 1996-1997: 24% [20%-29%]; 2004: 33% [28%-39%]) of deaths. Diarrhea was the second most important cause in 1993-1994 and 1996-1997, accounting for 11% (8%-14%) to 13% (9%-16%) of under-5 deaths. Birth asphyxia took over as the second important cause in 2004, claiming 13% (10%-17%) of all under-5 deaths.

### Trends in the cause-specific mortality rates

**Figure 3:**
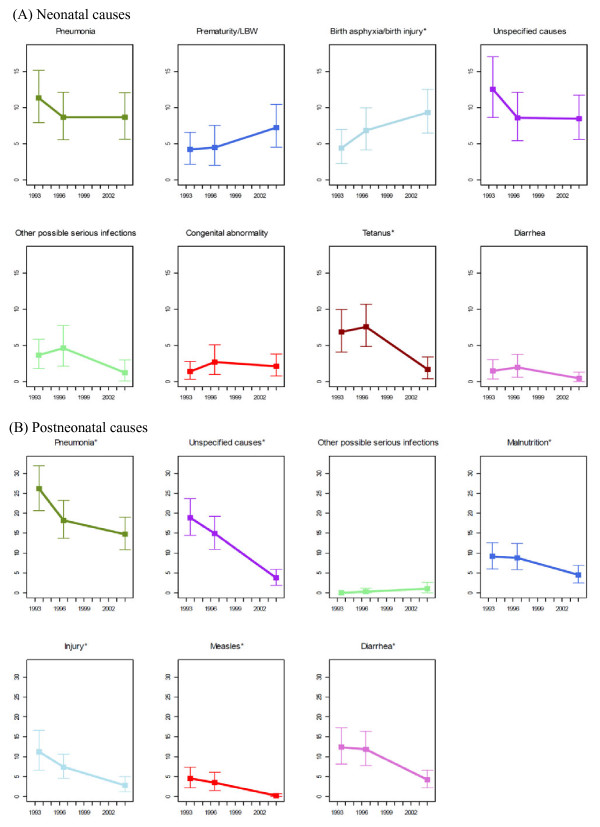
Cause-specific mortality rate of children under 5 years of age in Bangladesh, 1993-1994, 1996-1997, and 2004 (* indicates the change was statistically significant between 1993-1994 and 2004).

CSMRs and their uncertainty ranges are presented and compared in Figure 3 (also refer to Additional file 3 for the numeric values of these estimates). Among neonatal causes, the mortality rates of all causes dropped over the period with two exceptions: birth asphyxia/birth injury and prematurity/LBW. Birth asphyxia/birth injury increased from 4 (2-7) deaths per 1,000 live births (LB) in 1993-1994 to 7 (4-10) in 1996-1997 and then to 9 (6-13) per 1,000 LB in 2004. Prematurity increased from 4 (2-7) and 5 (2-8) deaths per 1,000 LB in the first two studies to 7 (4-11) deaths per 1,000 LB in 2004. The increase in birth asphyxia/birth injury between 1993-1994 and 2004 was statistically significant, but the increase was not significant for prematurity albeit the significant increase in its cause-specific fractions. Among declining causes, tetanus had the steepest drop between 1996-1997 and 2004, decreasing significantly from 7 (4-11) to 2 (0.4-4) per 1,000 LB. Other neonatal causes, such as pneumonia, diarrhea, and congenital abnormality, all showed a moderate decline in cause-specific mortality rates over the 10-year period, but the decline has not reached statistical significance.

Among deaths in the 1- to 59-month age group, the mortality rates of all of the causes dropped between 1993 and 2004, except for a slight increase in other possible serious infections, rising from 0 in 1993-1994 to 1 (0.1-2) per 1,000 LB in 2004 (Figure 3). The decline in cause-specific mortality rates among other causes was all significant. Pneumonia showed the fastest decline between 1993-1994 and 1996-1997, but reduced at a slower rate between 1996-1997 and 2004, dropping from 26 (20-33) to 15 (11-20) deaths per 1,000 LB between 1993 and 2004. Diarrhea showed the opposite pattern, staying relatively constant between the first two studies, but dropped the most quickly and significantly afterwards, declining from 12 (8-17) to 4 (2-7) deaths per 1,000 LB. The mortality rates of measles and injury decreased steadily and significantly from 5 (2-8) and 11 (7-17) to 0.2 (0-0.7) and 3 (1-5) per 1,000 LB, respectively. Malnutrition and unspecified causes dropped from 9 (6-13) and 19 (14-24) to 5 (2-7) and 4 (2-6) between 1993-1994 and 2004, respectively.

Among children younger than 5, the cause-specific mortality rates of pneumonia and diarrhea both dropped significantly, from 38 (31-46) to 23 (18-29) per 1,000 LB for pneumonia and from 14 (9-19) to 5 (2-7) per 1,000 LB for diarrhea. The top three causes changed from pneumonia, diarrhea, and injury in 1993-1994 (CSMR: 38 [31-46], 14 [9-19], and 11 [7-17] deaths per 1,000 LB, respectively), to pneumonia, birth asphyxia/birth injury, and prematurity in 2004 (CSMR: 23 [18-29], 9 [6-13] and 7 [4-11] deaths per 1,000 LB, respectively).

Our results are replicated in the sensitivity analysis, where deaths were first assigned to all possible causes without applying a hierarchy, and then multiple causes were assigned applying the standardized hierarchy. Most of the trends in CSMR are not statistically significant among neonates during the 10-year period, except those of birth asphyxia/birth injury and neonatal tetanus. However, postneonatal causes excluding other possible serious infections all saw significant declines in CSMR.

## Discussion

This study developed and applied a standardized computer-based algorithm to assign child causes of deaths using nationally representative verbal autopsy (VA) data in Bangladesh. The results provide distinctive insights into patterns and trends of child causes of death at the national level for one decade. The empirical trends are of particular interest as child causes of death are often modeled for most LMICs [1,2].

Our results corroborate the previous finding that pneumonia remains the top ranking cause of death among children below 5 years of age [1], despite the fact that the mortality rates of pneumonia had been declining significantly in Bangladesh. The proportional contribution of neonatal causes increased steadily during the study period. The proportion of under-5 deaths due to birth asphyxia/birth injury and prematurity/LBW rose progressively and significantly. The increase in their CSMR was also noticeable, although only the increase in the CMSR of birth asphyxia/birth injury reached statistical significance, possibly due to the competing significant reduction in the U5MR [4]. Likely because of a similar reason, the cause-specific fraction and the CSMR of neonatal tetanus both decreased, although only the reduction in CSMR was statistically significant. The composition of the top three ranking causes transitioned from including none neonatal cause to including two neonatal causes, which signifies again the increasing relative importance of neonatal mortality as under-5 mortality rate continues to decrease [15].

The CSMR of all postneonatal causes dropped significantly between 1993-1994 and 2004 except for other possible serious infections. Some of these changes, such as those in measles and diarrhea, may be partly explained by the increase in the coverage of measles vaccine and oral rehydration salts [7,11,12]. Other changes, however, have a less obvious association with changes in intervention coverage. For example, despite the significant decrease in the mortality rates of postneonatal pneumonia, the case management of pneumonia did not seem to improve [7,11,12], although access to high-quality, low-cost antibiotics is suggested to have been increasing during this period.

Several approaches are in widespread use to estimate the variability of population-level estimates for complex multistage sample survey data. The Jackknife and Balanced Repeated Replication (BRR) methods need multiple primary sampling units per sampling stratum [16]. These methods are both based on resampling of sample survey data. In this study, where each DHS sample had many levels of stratification, there were multiple strata where only one sampling unit was informative concerning the causes of death for children under 5. Resampling can accommodate stratification and clustering in multistage sampling data, and a more general approach relative to the Jackknife and BRR was used in our case, where an additional complexity was introduced due to the cause reallocation from the comorbidity of pneumonia and diarrhea to each individual cause as a nonlinear function of population estimates.

We developed a standardized algorithm to assign child causes of deaths using signs and symptoms collected through the VA studies to ensure comparability of the trends. The original algorithms applied in each study were systematically reviewed and key differences in cause categorization, hierarchy, case definition, and amount of data collected were compared to inform the development of the standardized algorithm. The standardized algorithm, primarily based on the 2004 cause categories and hierarchy, guarantees comparability of the trends by only including information commonly available across all studies. However, this was realized at the expense of losing additional information collected in the later studies.

When determining the cause categories, instead of mapping the causes to specific codes based on the ICD-10, we employed the CHERG cause categorization, which is in principle consistent with the ICD-10 rules. However, the CHERG classification does emphasize the relative public health importance of major child causes and encourages linkage with the relevant health interventions [9].

When presenting the final causes, we chose to combine possible pneumonia and possible diarrhea with pneumonia and diarrhea, respectively, for several reasons. First, based on the case definitions, we considered that possible infections were likely to be true cases of pneumonia/diarrhea with less severe symptoms. Second, after combining the possible diagnosis with the confirmed causes, the time trends were more stable. This practice is also consistent with previous approaches taken by CHERG [1,2]. However, the resulting trends in pneumonia need to be interpreted with caution. Further investigation shows that possible pneumonia is responsible for 12% to 26% of the deaths coded as pneumonia deaths among children aged 1 to 59 months old but 59% to 71% of the deaths coded as pneumonia among neonates. We suspect that the high proportion of neonatal possible pneumonia could be due to the inclusion of other serious infections, such as neonatal sepsis. As a common caveat of VA studies [17], additional misclassification errors may also exist in our results.

Comorbidity between diarrhea and pneumonia were reallocated among the two causes based on their relative importance in this study. Alternatively, cases with this type of comorbidity can be treated solely as pneumonia deaths. We took the former approach based on biological and medical considerations. However, more research is clearly needed to further determine which method is more appropriate. In fact, CHERG has an ongoing activity to examine the comorbidity patterns between pneumonia and diarrhea, which may contribute some knowledge to this area in the near future.

We estimated that malnutrition was responsible for 6% to 8% of under-5 deaths in Bangladesh in the study period. However, malnutrition could contribute to additional under-5 deaths as a risk factor [18]. In our sensitivity analysis where multiple causes were initially allowed, we found that comorbidity between malnutrition and any of the four infectious causes (diarrhea, ARI, measles, and other serious infections) exist among 42% to 55% of these deaths. Therefore, the estimated contribution of malnutrition to under-5 deaths needs to be interpreted carefully.

Several limitations are acknowledged regarding the present study. First, despite our attempts, several important causes, such as meningitis and neonatal sepsis, were not classified due to a lack of necessary symptom data in the two earlier studies. Moreover, unspecified causes contributed to 18% to 25% of under-5 deaths in Bangladesh between 1993 and 2004. It is suggested that more symptom data, especially relating to serious infections, should be routinely collected in future VA studies to facilitate ascertainment of additional causes.

The case definitions used herein were validated in Bangladesh, Nicaragua, and Uganda, and their validity has been shown to be reasonably good [10,19]. Similar hierarchies, though not validated, have been applied to the three Bangladesh VA studies originally and to a VA study conducted in parallel with the 2006 Nepal DHS [20]. Our standardized hierarchy, however, has the inherent limitations of any hierarchical process. In particular, the resulting cause-specific fractions are quite sensitive to the tier in which the causes were assigned [21,22]. For example, for comparison purposes, we assigned prematurity/LBW after other possible serious infections. The prematurity fractions were reduced by about 30% across all three studies. However, the hierarchy doesn't affect all deaths in the studies. In fact, our sensitivity analysis reveals that more than half (52%-54%) of the deaths were either due to a single cause or considered as belonging to "unspecified causes". Their cause fractions will remain the same irrespective of the application of hierarchies. Among the other half of the deaths with multiple diagnoses, many deaths could have been assigned to the same causes during medical certification.

Further validation research, such as the ongoing Grand Challenges in Global Health Initiative #13 Study done by the Population Health Metrics Research Consortium (PHMRC), may help determine a better computer-based algorithm. However, preliminary results of the PHMRC show that infectious causes, such as pneumonia, are still among the few causes that are hard to assign even after applying the advanced machine-learning theory. Whether these validated algorithms can be readily applied to secondary data is questionable since only limited information on signs and symptoms are available. In addition, the external validity of these algorithms in other countries and settings still needs to be shown.

Limited by the validity of the algorithm, the absolute level of the estimated cause-specific mortality rates may not be accurate. In other words, our uncertainty ranges did not take into account the unknown uncertainty in the cause of death assigning process. But with the employment of the standardized algorithm over the three datasets, the trends in the cause-specific mortality rates should be reasonably reliable. The uncertainties of the trends have been quantified by incorporating known uncertainties in the complex survey design and the U5MR. The values of these trends are further appreciated considering the fact that they originated from nationally representative empirical data. In the absence of a more reliable alternative, such information should start to be utilized to facilitate child health policymaking and resource allocation.

In the past, few empirical data on child causes of deaths have been available in LMICs, but recently more data are being collected and shared. Bangladesh is among a number of countries that have had at least one DHS with the VA module, and more countries are either planning on or considering including the VA component in their future DHSs. In addition, cause of death data collected through other sources, such as the International Network for the Demographic Evaluation of Populations and Their Health in Developing Countries (INDEPTH) and the Mozambique post-census survey applying the Sample Vital Registration using Verbal Autopsy (SAVVY) methodology, could all contribute to a better empirical understanding of cause of death [23]. Often, however, the heterogeneity in cause of death estimation could also be due to differences in methods for assigning or ascertaining causes [2,23]. Standardized algorithms are becoming an indispensible tool to generate child causes of death estimates that are comparable across time, countries, and settings [24]. Computer-based algorithms have the advantage of being objective, feasible, and affordable, and may better serve the needs of LMICs as compared to physician review. The current exercise is among the efforts to develop such standardized tools with the hope that more research of this type would be stimulated.

The resulting trends in child causes of death also provide a platform to link with trends in intervention coverage in Bangladesh. Close examination of these linkages, using packages like the Lives Saved Tool (LiST) [25], would help us better understand what drives the changes in cause-specific mortality and to identify mechanisms that work in the context of Bangladesh to reduce child mortality. These successful experiences or lessons learned can be shared with other countries to help accelerate their progress toward Millennium Development Goal 4.

## Conclusions

Despite a declining trend in the cause-specific mortality rate, pneumonia remained the top killer of children under 5 in Bangladesh from 1993 to 2004. The increasing importance of neonatal survival is highlighted by the growing contribution of neonatal deaths and several neonatal causes. Neonatal tetanus, birth asphyxia/birth injury, postneonatal pneumonia, diarrhea, measles, injury, and malnutrition all saw significant reductions in mortality rates, the driving factors of which still need to be better understood. Notwithstanding the limitations, standardized computer-based algorithms remain a promising tool to generate comparable child causes of death using VA data. The tool should prove particularly useful in places where routine medical certification is impractical yet information on the causes of death is regularly requested to improve child-survival planning. Repeated VA studies employing standardized instruments and algorithms would be especially appreciated for tracking trends in causes of child death.

## List of abbreviations

BDHS: Bangladesh Demographic and Health Survey; BRR: Balanced Repeated Replication; CHERG: Child Health Epidemiology Reference Group; CSMR: cause-specific mortality rates; IGME: the Inter-agency Group for Child Mortality Estimation; IHME: the Institute for Health Metrics and Evaluation; INDEPTH: International Network for the Demographic Evaluation of Populations and Their Health in Developing Countries; LB: live births; LBW: low birth weight; LiST: lives saved tool; LMIC: low- and middle-income country; NMR: neonatal mortality rate; SAVVY: Sample Vital Registration using Verbal Autopsy; U5MR: under-5 mortality rate; VA: verbal autopsy.

## Competing interests

The authors declare that they have no competing interests.

## Authors' contributions

LL developed the standardized algorithm, conducted the analysis, and wrote the first draft of this manuscript. QL assisted with data analysis. RAL helped review the original algorithms. IF contributed to the development of the standardized algorithm. JP assisted with the uncertainty estimation. REB and NW conceived the idea and supervise the study in general. All authors participated in results interpretation and subsequent revision of the manuscript.

## Supplementary Material

Additional file 1 **Case definitions of major child causes of death applied in the three Bangladesh VA studies and the standardized case definitions (differences in the case definitions between studies are underscored where applicable)**. Click here for file

Additional file 2 Cause-specific fractions and uncertainty ranges (in parentheses) in Bangladesh, 1993-1994, 1996-1997, and 2004 (* indicates the change was statistically significantly between 1993-1994 and 2004). Click here for file

Additional file 3 Cause-specific mortality rates (per 1,000 live births) and uncertainty ranges (in parentheses) in Bangladesh, 1993-1994, 1996-1997, and 2004 (* indicates the change was statistically significantly between 1993-1994 and 2004). Click here for file
