# Supplementary material for: Trends in causes of death among children under 5 in Bangladesh, 1993-2004: an exercise applying a standardized computer algorithm to assign causes of death using verbal autopsy data
Source: Popul Health Metr. 2011 Aug 5;9:43. doi: 10.1186/1478-7954-9-43 (PMC3160936; doi:10.1186/1478-7954-9-43)
Supplement: Additional file 1 — Case definitions of major child causes of death applied in the three Bangladesh VA studies and the standardized case definitions (differences in the case definitions between studies are underscored where applicable). [file 1478-7954-9-43-S1.PDF]

Additional file 1. Case definitions of major child causes of death applied in the three Bangladesh VA studies and the standardized case definitions (Differences in the case definitions between studies are underscored where applicable)

Cause	1993-1994 and 1996-1997	2004	Standardized case definitions
**Neonatal tetanus**	a. Age at death: 4-14 days;<br>AND<br>b. Convulsions;<br>AND<br>c1. Cried normally after birth but stopped crying <u>in the final illness</u>;<br>OR<br>c2. Suckled normally after birth but stopped suckling <u>in the final illness</u>;<br>OR<br>c3. <u>Both</u>	a. Age at death: 4-14 days;<br>AND<br>b. Convulsions;<br>AND<br>c1. Cried normally after birth but stopped crying <u>at least one day before the final illness</u>;<br>OR<br>c2. Suckled normally after birth but stopped suckling <u>at least one day before the final illness</u>	a. Age at death: 4-14 days;<br>AND<br>b. Convulsions;<br>AND<br>c1. Cried normally after birth but stopped crying <u>in the final illness</u>;<br>OR<br>c2. Suckled normally after birth but stopped suckling <u>in the final illness</u>
**Congenital abnormality**	a. Age at death: <=29 days;<br>AND<br>b. No cough;<br>AND<br>c. There was something physically wrong with the baby at birth	a. Age at death: <=28 days;<br>AND<br>b. The child had a reported malformation at birth	a. Age at death<=28 days;<br>AND<br>b1. There was something physically wrong with the baby at birth<br>OR<br>b2. The child had a reported malformation at birth
**Injury**	a. Injury including drowning	a. Age at death: >=29 days;<br>AND<br>b. accidental deaths including drowning	a. Age at death: >=29 days;<br>AND<br>b. accidental or injury deaths including drowning
**Birth asphyxia**	Diagnosed as "early neonatal deaths" where all deaths in the first 3 days of life were grouped together. They were not further sub-classified. However, symptom data needed to assign birth asphyxia were also collected.	a. Age at death: <=7 days;<br>AND<br>b. Not able to cry normally after birth;<br>AND<br>c1. Not able to breathe after birth;<br>OR<br>c2. Not able to suckle normally after birth	a. Age at death: <=7 days;<br>AND<br>b. Not able to cry normally after birth;<br>AND<br>c1. Not able to breathe after birth;<br>OR<br>c2. Not able to suckle normally after birth.
**Birth injury**	Same as above	a. Age at death: <=7 days;	a. Age at death: <=7 days;

		AND<br>b. Bruises or marks of injury on the body or head	AND<br>b. Bruises or marks of injury on the body or head
**Measles**	a. Age at death: >=6 months;<br>AND<br>b. Measles-type rash without water in the eruptions mostly on the face and trunk that appeared in 3 months before death;<br>AND<br>c. Accompanied by fever;<br>AND<br>d. With at least 1 of the following specific symptoms: dry cough, red or runny eyes, or running nose	a. Age at death: >=6 months;<br>AND<br>b. Measles-type rash without water in the eruptions all over the body and on the face that appeared in 3 months before death;<br>AND<br>c. Accompanied by fever;<br>AND<br>d. With at least 1 of the following specific symptoms: dry cough, red or runny eyes, or running nose	a. Age at death: >=6 months;<br>AND<br>b. Measles-type rash without water in the eruptions on body and fact that appeared in 3 months before death;<br>AND<br>c. Accompanied by fever;<br>AND<br>d. With at least 1 of the following specific symptoms: dry cough, red or runny eyes, or running nose
**Diarrhea**	Acute diarrhea<br>a. Frequent loose or liquid stools starting from 1 to 13 days before death and continuing until death;<br>AND<br>b. Without blood in the stool;<br>AND<br>c. With a peak number of 6 or more stools in 24 hours;<br>AND<br>d. With at least 2 of the 5 following specific symptoms: weakness, dry mouth, sunken eyes, depressed fontanels, or no or very little urine.<br><br>Dysentery was confirmed if all of the requirements for acute diarrhoea were met, with the additional requirement of blood in the stool.<br><br>Persistent diarrhoea was confirmed if	Neonatal diarrhea<br>a. Age at death<=28 days;<br>AND<br>b. Frequent liquid or watery or loose or soft stools or diarrhea was reported with a peak of 6 or more stools in 24 hours.<br><br>Postneonatal diarrhea<br>a. age at death: >=29 days;<br>AND<br>b. Frequent loose or liquid stools starting from 1 to 13 days before death and continued until death;<br>AND<br>c. With a peak number of 6 or more stools in 24 hours;<br>AND<br>d. With at least 2 of the 4 following specific symptoms: dry mouth, sunken eyes, loose skin, and no or very little urine.	Neonatal diarrhea<br>a. Age at death<=28 days;<br>AND<br>b. Frequent liquid or watery or loose or soft stools or diarrhea was reported with a peak of 6 or more stools in 24 hours.<br><br>Postneontal diarrhea<br>a. age at death:>=29 days;<br>AND<br>b. Frequent loose or liquid stools starting from 1 to 13 days before death and continued until death;<br>AND<br>c. With a peak number of 6 or more stools in 24 hours;<br>AND<br>d. At least 2 of the 6 following specific symptoms were reported: weakness, dry mouth, sunken eyes, loose skin, depressed fontanels, and no or very

	frequent loose or liquid stools started at least 14 days before death, and continued until death.	OR<br>e. Frequent loose or liquid stools started from at least 14 days prior to death and continued until death.	little urine.<br>OR<br>e. Frequent loose or liquid stools started from at least 14 days prior to death and continued until death.
**Acute Respiratory Infection (ARI)**	a1. Had a cough in the 2 weeks before death that started at least 3 days before death and lasted at least until the day before death;<br>OR<br>a2. Difficult breathing was reported in the 2 weeks before death, starting at least 1 day before death and lasted until death;<br>AND<br>b. Had at least 2 of the following 6 specific symptoms: noisy breathing, stridor, grunting, wheezing, nostril flaring or chest indrawing.	Neonatal ARI<br>a. Difficult or rapid breathing started at least 1 day before death and lasted until death;<br>AND<br>b. Had at least 2 of the following 3 specific symptoms: grunting, nostril flaring, and chest indrawing.<br><br>Postneonatal ARI<br>a1. Had a cough that started at least 3 days before death and lasted at least until the day before death;<br>OR<br>a2. Difficult or rapid breathing that started at least 1 day before death and lasted until death;<br>AND<br>b. Had at least 2 of the following 6 specific symptoms: noisy breathing, stridor, grunting, wheezing, nostril flaring, or chest indrawing.	Neonatal ARI:<br>a. Age at death<=28 days;<br>AND<br>b. Difficult breathing started at least 1 day before death and lasted until death;<br>AND<br>c. Had at least 2 of the following 3 specific symptoms: grunting, nostril flaring, and chest indrawing.<br><br>Postneonatal ARI:<br>a. Age at death>=29 days;<br>AND<br>b1. Had a cough that started at least 3 days before death and lasted at least until the day before death;<br>OR<br>b2. Difficult breathing that started at least 1 day before death and lasted until death;<br>AND<br>c. Had at least 2 of the following 6 specific symptoms: noisy breathing, stridor, grunting, wheezing, nostril flaring, or chest indrawing.

**Possible pneumonia**	Cough or difficult breathing	Neonatal possible pneumonia:<br>a. Age at death<=28 days;<br>AND<br>a. Had at least 2 of the following signs of serious infection: stopped suckling, stopped crying, difficult breathing, rapid breathing, chest<br>indrawing, convulsions, fever, cold to touch, lethargic, unresponsive or<br>unconscious, bulging fontanels, redness or drainage from the umbilical cord stump, skin rash with bumps<br>containing pus, or vomiting everything;<br>AND<br>c. difficult breathing or <u>rapid breathing</u><br><br>Postneonatal possible pneumonia:<br>a. Age at death>=29 days;<br>AND<br>b. Had at least 2 of the following signs of serious infection: difficult breathing, rapid breathing, chest indrawing, convulsions, fever, unresponsive or unconscious or stopped being able to grasp or stopped being able to respond to a voice or stopped being able to follow movements with the eyes, stiff neck, bulging fontanels, or vomiting;<br>AND<br>c. Cough or difficult breathing or <u>rapid breathing</u>	Neonatal possible pneumonia:<br>a. Age at death<=28 days;<br>AND<br>b. Had at least 2 of the following signs of serious infection: stopped suckling, difficult breathing, chest indrawing, convulsions, and fever.<br>AND<br>c. Difficult breathing<br><br>Postneonatal possible pneumonia:<br>a. Age at death>=29 days;<br>AND<br>b. Had at least 2 of the following signs of serious infection: difficult breathing, chest indrawing, convulsions, and fever;<br>AND<br>c. Cough or difficult breathing
**Possible diarrhea**	Loose or liquid stools	Neonatal possible diarrhea:<br>a. Age at death<=28 days;<br>AND<br>a. Had at least 2 of the following signs of serious infection: stopped suckling, stopped	Neonatal possible diarrhea:<br>a. Age at death<=28 days;<br>AND<br>b. Had at least 2 of the following signs of serious infection: stopped suckling, difficult

		crying, difficult breathing, rapid breathing, chest<br>indrawing, convulsions, fever, cold to touch, lethargic, unresponsive or<br>unconscious, bulging fontanels, redness or drainage from the umbilical cord stump, skin rash with bumps<br>containing pus, or vomiting everything;<br>AND<br>c. Loose or liquid stools<br><br>Postneonatal possible diarrhea:<br>a. Age at death>=29 days;<br>AND<br>b. Had at least 2 of the following signs of serious infection: difficult breathing, rapid breathing, chest indrawing, convulsions, fever, unresponsive or unconscious or stopped being able to grasp or stopped being able to respond to a voice or stopped being able to follow movements with the eyes, stiff neck, bulging fontanels, or vomiting;<br>AND<br>c. Loose or liquid stools	breathing, chest indrawing, convulsions, and fever.<br>AND<br>c. Loose or liquid stools<br><br>Postneonatal possible diarrhea:<br>a. Age at death>=29 days;<br>AND<br>b. Had at least 2 of the following signs of serious infection: difficult breathing, chest indrawing, convulsions, and fever;<br>AND<br>c. Loose or liquid stools
**Prematurity/Low Birth Weight**	a. Pregnancy lasted for <=7 months;<br>AND<br>b. The baby was reported to be much smaller than normal	a. Age at death<=28 days;<br>AND<br>b1. Pregnancy ended early<br>OR<br>b2. The baby was reported to be very small or smaller than usual	a. Age at death<=28 days;<br>AND<br>b1. Pregnancy ended early (<=7 months);<br>OR<br>b2. The baby was reported to be very small or smaller or much smaller than usual.

**Other possible serious infections**	Although other possible serious infections were not originally assigned, the following signs of serious infection were collected: stopped suckling, difficult breathing, chest indrawing, convulsions, and fever.	Neonatal other possible serious infections:<br>a. Age at death<=28 days;<br>AND<br>b. Had at least 2 of the following signs of serious infection: stopped suckling, stopped crying, difficult breathing, rapid breathing, chest<br>indrawing, convulsions, fever, cold to touch, lethargic, unresponsive or<br>unconscious, bulging fontanels, redness or drainage from the umbilical cord stump, skin rash with bumps<br>containing pus, or vomiting everything.<br><br>Postneonatal other possible serious infections:<br>a. Age at death>=29 days;<br>AND<br>b. Had at least 2 of the following signs of serious infection: difficult breathing, rapid breathing, chest indrawing, convulsions, fever, unresponsive or unconscious or stopped being able to grasp or stopped being able to respond to a voice or stopped being able to follow movements with the eyes, stiff neck, bulging fontanels, or vomiting.	Neonatal other possible serious infections:<br>a. Age at death<=28 days;<br>AND<br>b. Had at least 2 of the following signs of serious infection: stopped suckling, difficult breathing, chest indrawing, convulsions, and fever.<br><br>Postneonatal other possible serious infections:<br>a. Age at death>=29 days;<br>AND<br>b. Had at least 2 of the following signs of serious infection: difficult breathing, chest indrawing, convulsions, and fever.
**Malnutrition**	Very thin or the child’s feet were swollen	Very thin or the child’s feet were swollen	Very thin or the child’s feet were swollen
**Unspecified causes**	The remaining deaths	The remaining deaths	The remaining deaths
