# Supplementary material for: Trends in causes of death among children under 5 in Bangladesh, 1993-2004: an exercise applying a standardized computer algorithm to assign causes of death using verbal autopsy data
Source: Popul Health Metr. 2011 Aug 5;9:43. doi: 10.1186/1478-7954-9-43 (PMC3160936; doi:10.1186/1478-7954-9-43)
Supplement: Additional file 2 — Cause-specific fractions and uncertainty ranges (in parentheses) in Bangladesh, 1993-1994, 1996-1997, and 2004 (* indicates the change was statistically significantly between 1993-1994 and 2004). [file 1478-7954-9-43-S2.PDF]

Additional file 2. Cause-specific fractions and uncertainty ranges (in parentheses) in Bangladesh, 1993-1994, 1996-1997, and 2004 (* indicates the change was statistically significantly between 1993-1994 and 2004)

Age and cause	1993-1994	1996-1997	2004	Difference between 2004 and 1993-1994
**Neonates aged 0–27 days**				
Tetanus	0.054 (0.032 - 0.082)	0.069 (0.044 - 0.097)	0.024 (0.0066 - 0.049)	-0.030 (-0.061, 0.0030)
Congenital abnormality	0.011 (0.0025 - 0.022)	0.024 (0.010 - 0.044)	0.030 (0.012 - 0.050)	0.019 (-0.0030, 0.046)
Birth asphyxia/birth injury*	0.034 (0.016 - 0.056)	0.062 (0.038 - 0.093)	0.13 (0.089 - 0.18)	0.098 (0.054, 0.15)
Prematurity/LBW*	0.033 (0.016 - 0.054)	0.041 (0.019 - 0.069)	0.10 (0.063 - 0.16)	0.070 (0.027, 0.12)
Other possible serious infections	0.029 (0.014 - 0.048)	0.042 (0.021 - 0.067)	0.018 (0.0028 - 0.040)	-0.011 (-0.036, 0.017)
Unspecified causes	0.098 (0.066 - 0.14)	0.078 (0.051 - 0.11)	0.12 (0.077 - 0.17)	0.023 (-0.031, 0.078)
Diarrhea	0.012 (0.0025 - 0.023)	0.018 (0.0070 - 0.032)	0.0063 (0 - 0.017)	-0.0051 (-0.020, 0.011)
Pneumonia	0.088 (0.063 - 0.12)	0.078 (0.053 - 0.11)	0.12 (0.086 - 0.17)	0.035 (-0.016, 0.091)
**Children aged 1–59 months**				
Injury*	0.088 (0.052 - 0.13)	0.067 (0.041 - 0.094)	0.039 (0.016 - 0.077)	-0.048 (-0.096,-0.0010)
Measles*	0.035 (0.017 - 0.060)	0.031 (0.015 - 0.054)	0.0028 (0 - 0.0092)	-0.033 (-0.055, -0.014)
Other possible serious infections	0	0.0028 (0 - 0.0085)	0.015 (0.0011 - 0.035)	0.015 (0.000, 0.038)
Malnutrition	0.071 (0.046 - 0.10)	0.080 (0.051 - 0.11)	0.064 (0.034 - 0.10)	-0.0070 (-0.047, 0.034)
Unspecified causes*	0.15 (0.11 - 0.19)	0.14 (0.097 - 0.18)	0.053 (0.027 - 0.085)	-0.094 (-0.14, -0.048)
Diarrhea	0.096 (0.064 - 0.13)	0.11 (0.074 - 0.14)	0.060 (0.032 - 0.091)	-0.037 (-0.084, 0.010)
Pneumonia	0.20 (0.16 - 0.25)	0.16 (0.12 - 0.20)	0.21 (0.16 - 0.26)	0.0050 (-0.066, 0.076)
**Children aged 0–59 months**				
Diarrhea	0.11 (0.075 – 0.14)	0.13 (0.092 – 0.16)	0.066 (0.037 – 0.098)	-0.042 (-0.090, 0.0070)
Pneumonia	0.29 (0.25 – 0.34)	0.24 (0.20 – 0.29)	0.33 (0.28 – 0.39)	0.04 (-0.039, 0.12)
All neonatal causes*	0.36 (0.31 – 0.41)	0.41 (0.37 – 0.46)	0.56 (0.49 – 0.62)	0.20 (0.12, 0.29)
All causes	1	1	1	
