# Supplementary material for: Trends in causes of death among children under 5 in Bangladesh, 1993-2004: an exercise applying a standardized computer algorithm to assign causes of death using verbal autopsy data
Source: Popul Health Metr. 2011 Aug 5;9:43. doi: 10.1186/1478-7954-9-43 (PMC3160936; doi:10.1186/1478-7954-9-43)
Supplement: Additional file 3 — Cause-specific mortality rates (per 1,000 live births) and uncertainty ranges (in parentheses) in Bangladesh, 1993-1994, 1996-1997, and 2004 (* indicates the change was statistically significantly between 1993-1994 and 2004). [file 1478-7954-9-43-S3.PDF]

Additional file 3. Cause-specific mortality rates (per 1,000 live births) and uncertainty ranges (in parentheses) in Bangladesh, 1993-1994, 1996-1997, and 2004 (* indicates the change was statistically significantly between 1993-1994 and 2004)

Age and cause	1993-1994	1996-1997	2004	Difference between 2004 and 1993-1994
**Neonates aged 0–27 days**				
Tetanus*	6.9 (4.1 - 10.6)	7.6 (4.9 - 10.7)	1.7 (0.4 - 3.5)	-5.2 (-8.7, -2.1)
Congenital abnormality	1.4 (0.3 - 2.8)	2.7 (1.1 - 4.8)	2.1 (0.8 - 3.9)	0.7 (-1.2, 2.9)
Birth asphyxia/birth injury*	4.4 (2.1 - 7.2)	6.8 (4.2 - 10.3)	9.3 (6.3 - 12.8)	4.9 (1.0, 8.8)
Prematurity/LBW	4.2 (2.1 - 6.9)	4.5 (2.1 - 7.6)	7.2 (4.4 - 10.9)	3.0 (-0.6, 6.8)
Other possible serious infections	3.7 (1.8 - 6.1)	4.6 (2.2 - 7.7)	1.2 (0.2 - 2.8)	-2.4 (-5.0, 0.2)
Unspecified causes	12.5 (8.4 - 17.7)	8.6 (5.6 - 12.3)	8.5 (5.4 - 12.2)	-4.1 (9.4, 1.1)
Diarrhea	1.5 (0.3 - 3.0)	2.0 (0.7 - 3.7)	0.4 (0 - 1.3)	-1.0 (-2.8, 0.3)
Pneumonia	11.3 (7.8 - 15.5)	8.7 (5.6 - 12.6)	8.7 (5.6 - 12.6)	-2.7 (-3.1, -2.7)
**Children aged 1–59 months**				
Injury*	11.2 (6.7 - 17.1)	7.4 (4.5 - 10.4)	2.8 (1.1 - 5.4)	-8.5 (-14.3, -3.3)
Measles*	4.5 (2.1 - 7.7)	3.5 (1.7 - 6.0)	0.2 (0 - 0.7)	-4.3 (-7.2, -2.1)
Other possible serious infections	0	0.3 (0 - 1.0)	1.0 (0.1 - 2.4)	1.0 (0.0, 2.7)
Malnutrition*	9.1 (5.9 - 12.9)	8.8 (5.6 - 12.5)	4.5 (2.4 - 7.1)	-4.6 (-8.7, -0.8)
Unspecified causes*	18.9 (14.1 - 24.3)	14.9 (10.7 - 19.8)	3.7 (1.9 - 6.0)	-15.1 (-20.3, -10.2)
Diarrhea*	12.4 (7.9 - 17.0)	11.9 (7.8 - 16.6)	4.2 (2.1 - 6.8)	-8.2 (-13.5, -3.5)
Pneumonia*	26.2 (20.0 - 32.9)	18.2 (13.2 - 23.6)	14.7 (10.6 - 19.8)	-11.5 (-18.4, -4.3)
**Children aged 0–59 months**				
Diarrhea*	13.8 (9.3 – 18.9)	13.8 (9.7 – 18.8)	4.7 (2.5 – 7.4)	-9.2 (-14.6, -4.3)
Pneumonia*	37.5 (30.6 – 45.5)	26.9 (20.9 – 33.2)	23.4 (18.1 – 29.4)	-14.1 (-22.0, -6.0)
All neonatal causes	45.9 (38.1 – 54.4)	45.5 (38.7 – 53.1)	39.2 (32.3 – 46.3)	-6.7 (-15.2, 2.1)
All causes	128.2	110.4	70.3	
